# Self-Assembly of Supramolecular Gels in Complex Anti-icing Fluids to Create Multicomponent Materials with Enhanced Performance

**DOI:** 10.1021/acs.langmuir.5c05067

**Published:** 2025-12-05

**Authors:** Nicole K. McLeod, Lee Stokes, Jerry Lewis, David K. Smith

**Affiliations:** † Department of Chemistry, 8748 University of York, Heslington, York YO10 5DD, U.K.; ‡ 375896 Kilfrost Ltd, Albion Works, Haltwhistle NE49 0HJ, U.K.

## Abstract

Low-molecular-weight gelators (LMWGs) based on 1,3:2,4-dibenzylidenesorbitol (DBS, DBS-OCH_3_ and DBS-SCH_3_) were formulated into anti-icing products (ABC 3, ABC K+, ABC S+) containing polymeric additives, creating hybrid gels. Gels formed at significantly lower concentrations than in the base solvent (monopropylene glycol:H_2_O, 50:50), suggesting that the LMWG and the polymer in the anti-icing fluid can cooperate to form a sample-spanning gel. More thermodynamically stable, stiffer gels assemble more rapidly in higher-performance anti-icing fluids (ABC S+ and ABC K+). This suggests that LMWG-polymer interactions in lower-performance fluids (ABC 3) may compete with the assembly of the LMWG, reducing its ability to form a sample-spanning network. TEM visualized nanoscale fibrillar networks for all LMWGs in these anti-icing fluids. In the case of the most hydrophobic gelator (DBS-SCH_3_), globular structures associated with the polymeric additive were attached to these gel fibers. This correlated with the fact that DBS-SCH_3_ gave the softest, least stable gels suggestive of interactions between the LMWG and the polymer. The LMWG-modified anti-icing fluids were tested as anti-icing agents and significant increases in “holdover time” were achieved. As expected based on our observations of gel assembly, these increases were least significant in ABC 3. Pleasingly, Type II anti-icing fluid ABC K+ could be improved to a high-performance Type IV fluid by the addition of very small amounts of these LMWGs (0.25 g/L). Aerodynamic testing indicated that the gels were broken down by strain. Given the low cost of these LMWGs, the ease of coformulation by simple mixing, and the significant enhancements in anti-icing performance, we suggest they may see application in this technology.

## Graphical abstract

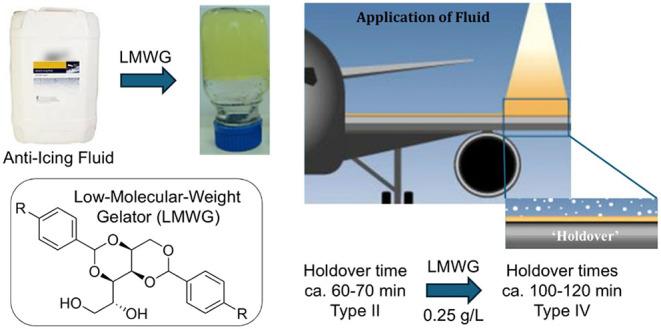

## Introduction

Low-molecular-weight gelators (LMWGs) that self-assemble into supramolecular gels^1,2^ are versatile systems with a variety of industrial applications, including in areas such as viscosity modification,^3^ adhesives,^4^ and personal care.^5^ Beyond established industrial use, there is burgeoning interest in the academic development of these tunable soft materials, with one eye on potential future high-tech applications.^6,7^

An area of recent academic interest has been the combination of self-assembling LMWGs with polymer technology.^8^ Indeed, such work builds on established industrial uses of LMWGs in controlling the polymer melt phase to achieve transparent plastics,^9–12^ as well as their uses in dentistry^13,14^ and 3D printing,^15,16^ where an *in situ* self-assembled LMWG network helps control photopolymerization. Given that supramolecular gels depend on reversible noncovalent interactions, they are often inherently weak materials. Combining LMWGs with polymer gels is therefore an established strategy for enhancing rheological performance.^8^ For example, LMWGs have been mixed with the polymer gelator agarose to provide greater robustness.^17^ Alternatively, supramolecular gels have been shaped into easily handled core–shell bead-like objects by formulation with the polymer gel calcium alginate.^18^

There is considerable current interest in multicomponent supramolecular gels, in which LMWGs are combined with other systems.^19,20^ It is possible that individual components can: (i) self-sort and form their own nanoscale networks, (ii) coassemble and form a new nanoscale system, or (iii) disrupt one another’s assembly/performance.^21^ The outcome in any specific case depends on the strengths of interactions between different components, their preferred assembly pathways in the relevant environment, and the “history” of treatment to which they have been exposed.^22^

One particularly interesting application of modified gel-like fluids is in anti-icing—of great importance in a variety of settings, for example, aviation.^23,24^ There are four different types of deicing and anti-icing agent (Types I–IV) for use on aircraft, each with a different purpose.^25^ Type I products are simple deicing agents based on glycol/water mixtures with surfactant additives and have low viscosity—they are designed to remove ice from a frozen surface. Type II–IV products are anti-icing agents that actively prevent ice buildup while the aircraft is waiting to take off. Type II products contain a pseudoplastic thickening polymer in a fluid with a minimum glycol content of 50%, which creates a film, providing “holdover protection” to an aircraft—a period of time in which ice should not reform on treated surfaces. The film is ultimately removed by the shear forces associated with aircraft takeoff. Type III fluids are similar but designed for use on aircraft with lower takeoff speeds—they typically have lower holdover times. Type IV products are the highest-performance anti-icing agents and use different polymeric thickening agents that significantly extend holdover times, allowing aircraft to have more time between treatment and takeoff.

Beyond industrial application, there is also growing academic interest in anti-icing technology, with attention starting to focus on polymer gels as next-generation anti-icing agents.^26^ In spite of the dominance of polymer technology in the field,^27–33^ low-molecular-weight gelators (LMWGs) potentially have a lot to offer in this type of application. LMWGs have recently been investigated as cryopreservants to protect cells from the crystallization of ice using their self-assembled networks.^34–36^ LMWGs are well-suited to act as industrial anti-icing agents, as they can form at low loading levels and have a high degree of sensitivity to shear. However, although there have been sporadic reports of LMWGs that assemble into supramolecular gels in water/glycol mixtures,^37–39^ there had been no attempts to optimize LMWGs for anti-icing applications until our own very recent work.^40^ In this previous work, we used gelators based on 1,3­(*R*):2,4­(*S*)-dibenzylidene-d-sorbitol (DBS, Figure 1A) in mixtures of monopropylene glycol (MPG) and water (Figure 1B) and demonstrated effective gel formation. DBS self-assembles via intermolecular hydrogen bond interactions between the sorbitol groups, and the solvophobic effect/π-π stacking of the aromatic rings, the chirality of which is defined by thermodynamic control during LMWG synthesis.^41^ The balance between interactions depends on the solvent,^42–44^ giving this class of gelator broad scope, immobilizing a wide range of organic solvents.^45–48^ On formulating these LMWGs into a Type I aviation deicing fluid, “DF+”,^40^ we then demonstrated that the holdover times were significantly improved—indeed, in some cases (depending on LMWG loading and solvent composition), the simple deicing fluid behaved more like a Type III anti-icing fluid (i.e., the holdover time increased from >3 min to >20 min under standard conditions, Figure 1D). Furthermore, the gels were effectively broken down by shear forces equivalent to aircraft takeoff, meeting the required standard for aerodynamic compatibility.

**Figure 1:**
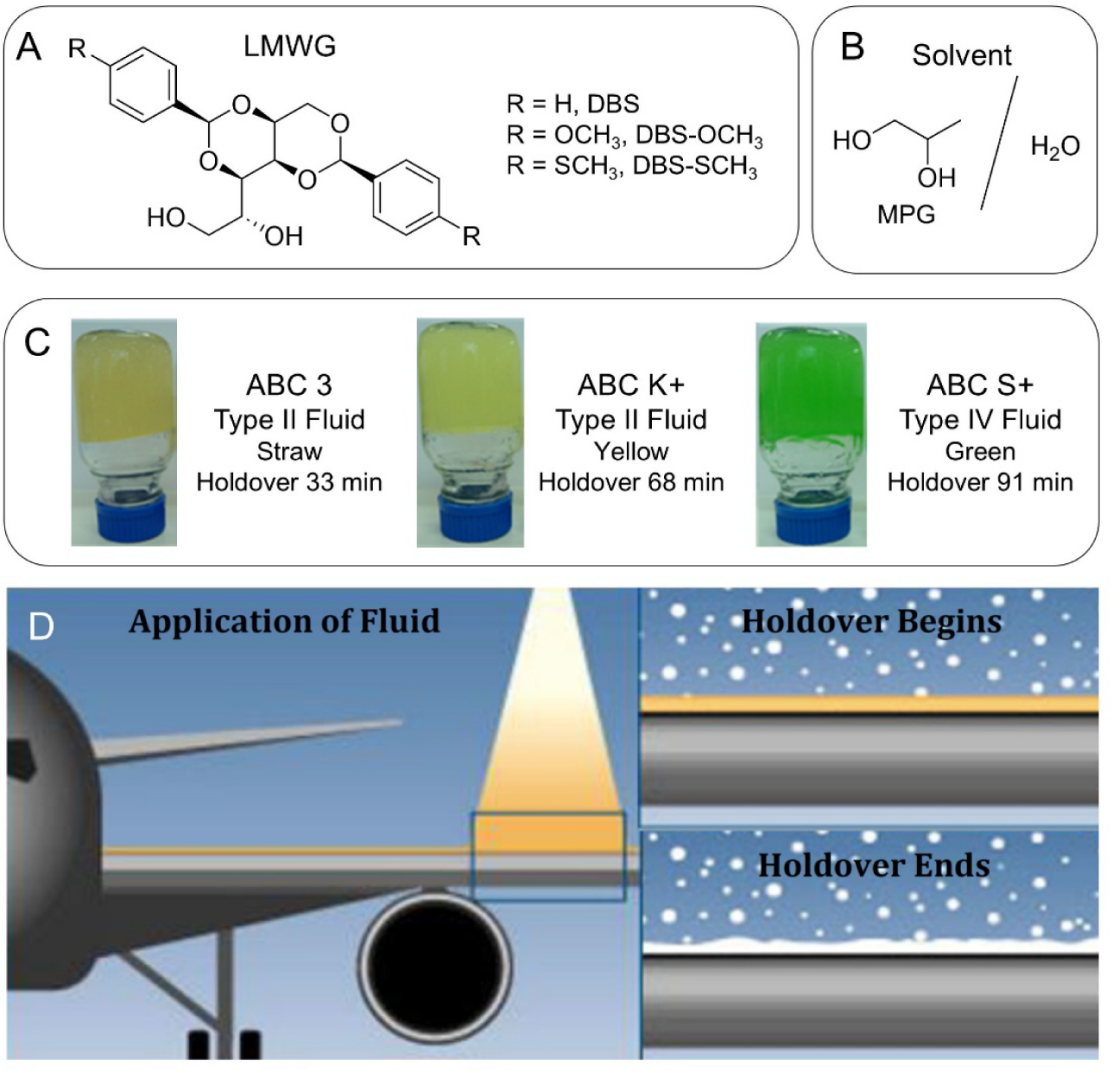
(A) Structures of 1,3­(*R*):2,4­(*S*)-dibenzylidenesorbitol (DBS) low-molecular-weight gelators (LMWGs) that were investigated in this study. (B) The solvent used in anti-icing agents is based on a 50:50 mixture of monopropylene glycol (MPG) and water. (C) Inverted bulk gel samples were made in commercial anti-icing fluids ABC 3, ABC K+, and ABC S+. (D) Schematic showing anti-icing fluid applied to an aircraft wing, indicating the holdover effect, which ends once the surface is no longer free of ice.

Given the promise of these results, we decided to investigate LMWGs as additives in more complex Type II and IV anti-icing fluids (Figure 1C). These fluids are based on MPG:H_2_O (50:50) but also contain a polymer to provide much-enhanced holdover time performance, as well as a variety of other additives, including neutral nonionic surfactants. These systems can therefore be considered as multicomponent materials. In this respect, it is possible that the LMWG may self-assemble in its usual manner, behaving independently of the remainder of the fluid and hence simply adding its own rheological anti-icing performance to the whole. However, it is also possible that the LMWG may interact with components of the fluid, disrupting it and modifying its performance in different ways.

## Experimental Section

### Materials

1,3:2,4-Dibenzylidene-d-sorbitol was purchased from Rika International, commercially named “Geniset D” and was used without further purification. DBS-OCH_3_ and DBS-SCH_3_ were synthesized as described previously, and all characterization data were in agreement with previous reports.^40,49^ The commercial anti-icing products ABC 3, ABC K Plus ('ABC K+'), and ABC S Plus ('ABC S+') were provided by Kilfrost Limited and used as supplied.

### Preparation of Gels

#### Preparation of 1 mL Gel Samples Using Anti-icing Fluids

Samples were prepared by accurately weighing the solid gelator into a 2 mL glass vial and adding 1 mL of solvent. Type II (ABC 3 and ABC K+) and Type IV (ABC S+) products were used as the solvent without being diluted. Each sample was then sonicated for 1 h before being heated in an oil bath to below the boiling point of water until clear, homogeneous solutions were formed. If, after 1 h of heating, the sample did not form a clear, homogeneous solution, it was removed from the heat. The samples were left on the bench at room temperature overnight, during which time they formed gels. Results were recorded the next day.

#### Scaling Up Gel Samples in Anti-icing Fluids

A known mass of LMWG was weighed into a glass Schott bottle, and 200 mL of solvent (Type II and Type IV anti-icing products) was added. Samples were sonicated for 1 h before being heated in an oil bath to below the boiling point of the solvent until a clear, homogeneous solution was formed—they were normally left overnight. Samples were then removed from the heat and left to cool under ambient conditions to allow the gels to form. Duplicate samples were made.

#### Dilution of Gels for Testing as Anti-icing Agents

Gels formed as described above were also diluted to 50% for testing with water spray endurance. Samples at 50% dilution (100 mL) for each of the anti-icing product gels were made by transferring the gel sample (50 mL) to a measuring cylinder with the addition of hard (tap) water (50 mL). The samples were mixed by inverting the measuring cylinder. These samples were left at room temperature prior to testing.

#### Characterization of Gels

Gel characterization was performed by measuring minimum gelation concentration and thermal stability, performing rheology, electron microscopy, and NMR, and using industry-standard methods like water spray endurance testing (WSET). The methods were all performed as in our previously published work^40^ and are described fully in the Supporting Information.

## Results and Discussion

### Low-Molecular-Weight Gelators (LMWGs)

We selected DBS, DBS-OCH_3_, and DBS-SCH_3_, as they had been demonstrated to be optimal for forming supramolecular gels in mixtures of monopropylene glycol (MPG) and water in our previous work.^40^ DBS was obtained from a commercial source, while DBS-OCH_3_ and DBS-SCH_3_ were synthesized as described previously.^40,49^

### Anti-icing Agents

We decided to work with three different anti-icing agents produced by Kilfrost—ABC 3, ABC K+, and ABC S+. Of these fluids, ABC 3 and ABC K+ are Type II anti-icing fluids and are colored straw and yellow, respectively, with ABC K+ being the standard product designed for use in more challenging snow conditions. ABC S+ is a high-performance Type IV anti-icing fluid with maximum holdover performance, also suitable for use in snow conditions, and is colored green. Each of these anti-icing products has MPG:H_2_O (50:50) as the base solvent. Anti-icing products are commonly used in their original undiluted form but can sometimes be diluted with water to “75%” (37.5:62.5, MPG:H_2_O) and “50%” (25:75, MPG:H_2_O) by consumers if desired. The precise composition of these anti-icing fluids is proprietary information and therefore not fully disclosed here. Information on the general types of additives present in anti-icing agents can be found in documentation from the aviation industry.^52^ However, all three fluids used here are based on variations of a similar polymeric film-forming ingredient. The performance of anti-icing products can typically be tuned by changing features such as polymer composition, choice of monomers/comonomers, type and degree of cross-linking, giving rise to anti-icing products with different levels of performance. In each case, the polymer is present at a relatively low loading of <1% wt/vol in the finished anti-icing product.

### Gel Testing—Initial “Tabletop” Studies

Initially, we wanted to determine whether our LMWGs could form gels directly in these anti-icing fluids. In our previous work,^40^ the deicing fluid DF+ was based on 80:20 MPG:H_2_O, whereas these products are based on 50:50 MPG:H_2_O and contain significantly more additives. It was therefore important to determine whether the LMWGs could still self-assemble in these more complex anti-icing fluids. We weighed out a known amount of LMWG into a 2 mL glass vial and added anti-icing fluid (1 mL). Each sample was sonicated for 30 min before being heated in an oil bath at 90 °C until a clear homogeneous solution was formed. Samples were removed from the heat and left on the bench overnight.

Pleasingly, homogeneous transparent gels could be formed by DBS directly in the anti-icing products (Figure 2, Figure S1). At high concentrations (1% or 2% wt/vol), DBS remained partly insoluble, and transparent gels were not formed—this insolubility results from the relatively high water content of these products combined with the high LMWG loading. At lower concentrations (0.1%–0.5% wt/vol), DBS dissolved fully, and transparent gels were obtained. At very low concentrations (0.05% wt/vol), partial gels were formed, which collapsed on inversion. The minimum gelation concentration (MGC) was ca. 0.06% wt/vol. DBS-OCH_3_ behaved similarly to DBS (Table S1). In contrast, DBS-SCH_3_ showed somewhat different behavior. This LMWG failed to fully dissolve even at loadings of 0.5% and 1.0% wt/vol and hence did not form effective gels. This is in line with our previous findings that this gelator is more hydrophobic and has lower solubility in the presence of significant amounts of water.^40^ However, sample-spanning gels were formed at low concentrations with an MGC value of 0.04% (Table S1). A partial gel was even formed at 0.025% wt/vol. This suggests a greater ability of DBS-SCH_3_ to form gels at low loading as a result of its more hydrophobically driven assembly, again in agreement with our previous work.^40^ As described in the Introduction, these LMWGs primarily assemble as a result of hydrogen bonding and solvophobic interactions between the sorbitol ‘bodies and solvophobic π-π stacking between aromatic rings. The primary role of the substituents is not to introduce new LMWG–LMWG interactions but rather to tune the overall interactions of the LMWG with the solvent. The hydrophobically driven assembly of DBS-SCH_3_ in comparison to DBS-OCH_3_ can therefore be understood in terms of the differences between sulfur and oxygen. As an example of this, considering the Kamlet–Taft parameters of diethyl ether and diethyl sulfide, the α value for hydrogen bond acceptance drops from 0.47 to 0.37, while the π* value for polarizability increases from 0.27 to 0.46. As such, the lower hydrogen bonding and greater polarizability of DBS-SCH_3_ compared with DBS-OCH_3_ will increase its hydrophobicity, helping drive self-assembly in these mixed aqueous solvents. As noted in our previous work,^40^ it is also possible that chalcogen-chalcogen interactions may help reinforce assembly in DBS-SCH_3_.

**Figure 2:**
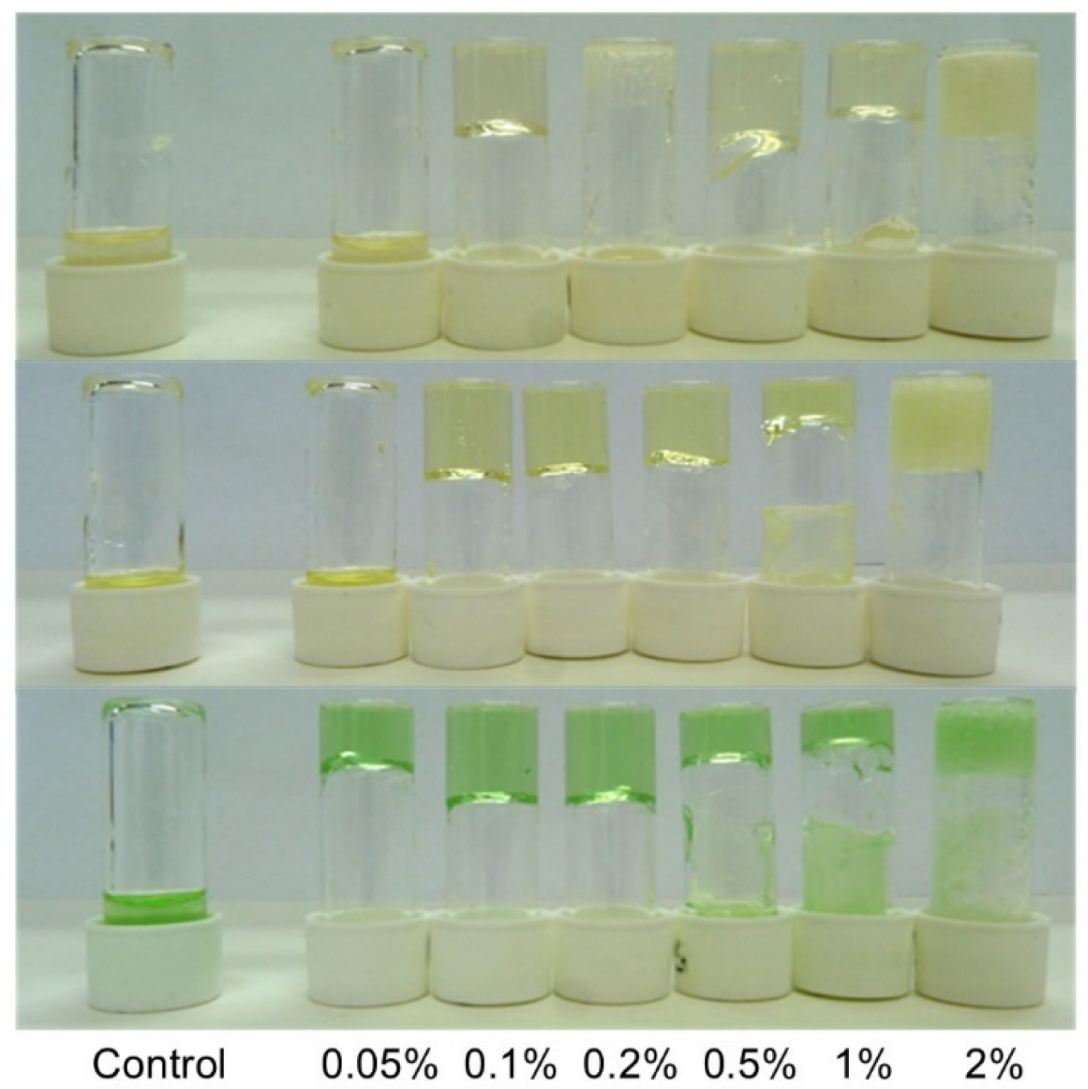
Photographs of gels formed by DBS in the anti-icing fluids ABC 3 (top), ABC K+ (center), and ABC S+ (bottom) at different loadings from 0.05% to 2% wt/vol.

As a control experiment, samples of each anti-icing product without LMWG present were also treated with sonication and a heat–cool cycle to ensure the polymeric additives in the fluids did not themselves become gel-forming—all remained as clear, viscous solutions, proving that the LMWG is responsible for gel formation.

It is notable that, in these anti-icing fluids, the MGC values of DBS and DBS-OCH_3_ are lower than the MGCs previously determined for these LMWGs in MPG:H_2_O (50:50) (DBS 0.07% wt/vol, DBS-OCH_3_ 0.10% wt/vol).^40^ We suggest that this may be a result of the polymeric additive present in Types II and IV anti-icing fluids. This polymer plays an active role in viscosity modification in these fluids, and although it does not itself form a gel, it is realistic to reason that the self-assembled LMWG network and polymer have combined effects, potentially enabling gel formation at lower loadings, with the PG acting as a scaffold that facilitates the assembly of the LMWG, meaning it does not have to span such wide spaces. For DBS-SCH_3_ there is not the same improvement in MGC, which might suggest the LMWG is already operating at the limits of performance, or that it does not benefit from the presence of the polymer in the same way (see discussion below).

Thermal stabilities (*T*_gel_ values, Figure S2) were then determined using the tube inversion methodology (see below for more detailed temperature-dependent rheological characterization). For DBS, the *T*_gel_ value was identical irrespective of the anti-icing fluid being used (*T*_gel_ at 0.1% wt/vol, 59 °C) and showed the expected trend of increasing with increasing LMWG loading. Interestingly, the thermal stability was higher than that previously observed in DF+ Type 1 deicing fluid diluted to 50% MPG content (*T*_gel_ at 0.1% wt/vol, 41 °C).^40^ At higher loadings, the difference narrowed but remained at ca. 5 °C. This suggests that in the ABC-type anti-icing fluids, the presence of the polymer enhances the thermal stability of the gel. Very similar *T*_gel_ values and trends in the different products were observed for DBS-OCH_3_. Again, the thermal stability was greater in the anti-icing fluids (*T*_gel_ at 0.1% wt/vol, 60–65 °C) than in DF+ diluted to 50% MPG (*T*_gel_ at 0.1% wt/vol, 41 °C). DBS-SCH_3_ had a similar *T*_gel_ value to the other LMWGs at a loading of 0.1% wt/vol in the anti-icing fluids (60–65 °C), which decreased as the loading was lowered, as would be expected.

### Gel Rheology

Rheological analysis of each of the anti-icing products was performed and compared to that of each of the LMWGs in each of the anti-icing products. In the absence of LMWG, ABC 3 has a *G*′ value of 2.7 Pa and a critical strain (*G*′ = *G*″) value of ca. 0.6%. On addition of each of the LMWGs (0.1% wt/vol), the *G*′ increased a little (*G*′ = 3–10 Pa), while the critical strain value remained about the same (Table 1, Figure 3A, Figures S4–S5). The *G*′ values increased in the order DBS-OCH_3_ < DBS-SCH_3_ < DBS. The increased stiffness reflects the LMWG acting to somewhat stiffen the polymeric product. Although soft, weak gels were formed, variable frequency studies showed that they were only independent of frequency up to about 1 Hz.

**Figure 3:**
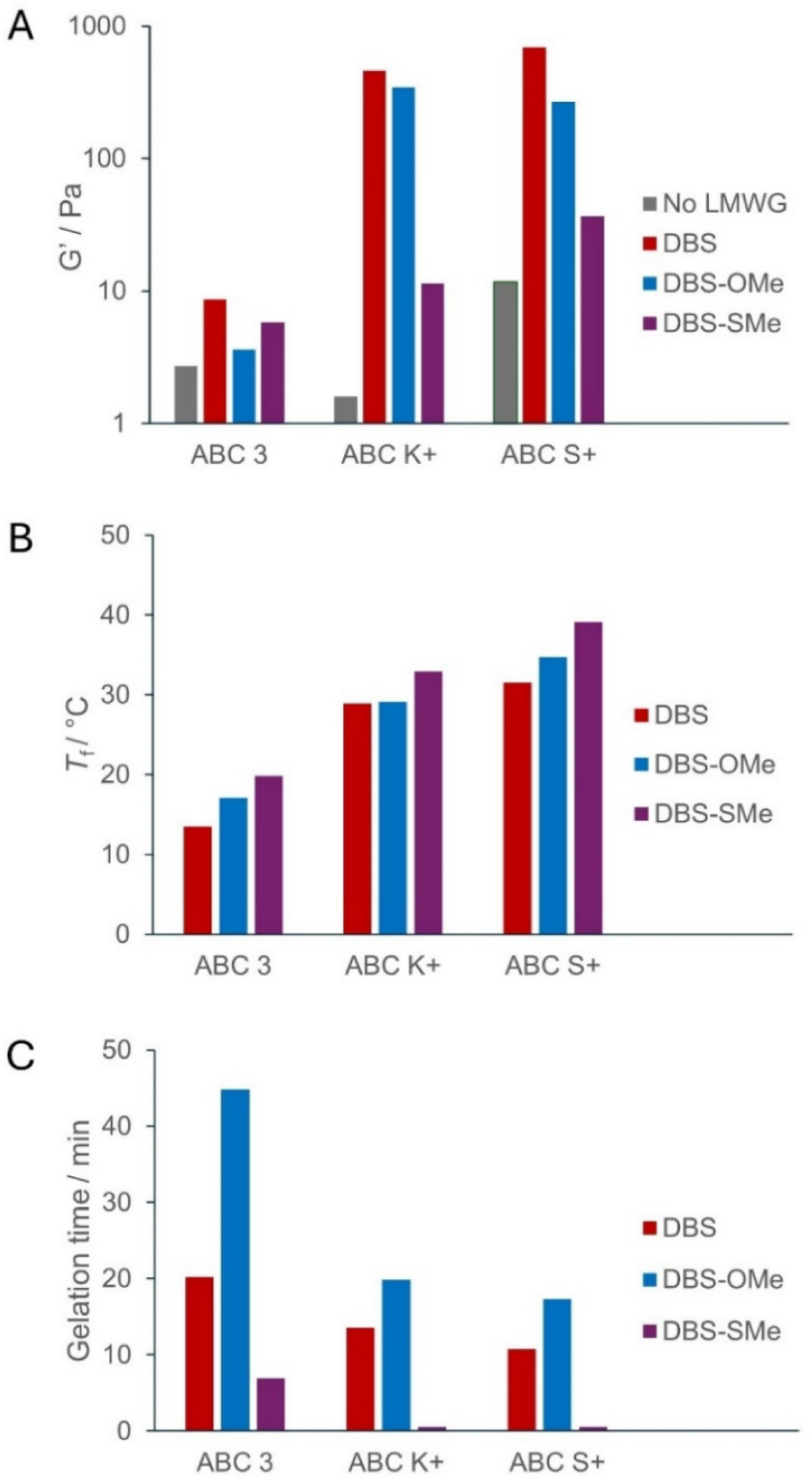
(A) *G*′ values (Pa) of anti-icing fluids and gels formed in them at LMWG loading of 0.1% wt/vol. (B) *T*_f_ values (°C) of gels formed in anti-icing fluids at LMWG loading of 0.1% wt/vol. (C) Time required for gelation (min) in anti-icing fluids at LMWG loading of 0.1% wt/vol.

**Table 1:** Rheological Characterization of DBS, DBS-OCH_3_, and DBS-SCH_3_ in Anti-icing Products ABC 3, ABC K+, and ABC S+ at a Loading of 0.1% wt/vol

		No LMWG	DBS	DBS-OCH_3_	DBS-SCH_3_
ABC 3	*G*′/Pa	2.7	8.6	3.6	5.8
	*G*′ = *G*″/%	0.6	0.6	0.5	0.6
	Frequency threshold/Hz	-	1	1	1
ABC K+	*G*′/Pa	1.6	460	347	11.3
	*G*′ = *G*″/%	1.6	3.2	3.2	0.6
	Frequency threshold/Hz	-	15	15	2.5
ABC S+	*G*′/Pa	11.7	603	268	36.7
	*G*′ = *G*″/%	2.6	2.5	3.2	0.8
	Frequency threshold/Hz	1	15	15	8

When formulated into ABC K+, the LMWGs (0.1% wt/vol) had a much larger effect on stiffness (Table 1, Figure 3A, Figures S4–S5). The product itself had a *G*′ value of 1.6 Pa. This increases to 11, 347, and 460 Pa with DBS-SCH_3_, DBS-OCH_3_, and DBS respectively. Furthermore, for DBS-OCH_3_ and DBS the critical strain increases from 1.6% to 3.2%. Variable frequency studies indicated gel-like behavior that was independent of frequency to ca. 15 Hz for the stiffer gels formed by DBS and DBS-OCH_3_. The softer gel formed by DBS-SCH_3_ was independent of frequency to ca. 2.5 Hz.

When the LMWGs were incorporated into the Type IV anti-icing product, ABC S+, once again the stiffness of the gel network increased significantly (Table 1, Figure 3A, Figures S4–S5). The product itself exhibits a *G*′ value of 12 Pa. This increases to 37, 268, and 603 Pa with DBS-SCH_3_, DBS-OCH_3_, and DBS respectively. Variable frequency studies indicated gel-like behavior that was independent of frequency up to ca. 15 Hz for the stiffer gels formed by DBS and DBS-OCH_3_. The softer gel formed by DBS-SCH_3_ was independent of frequency up to about 8 Hz.

It is, therefore, evident that the LMWGs exhibit a degree of stiffening in all gels. This effect is least marked for ABC 3 and most significant for DBS and DBS-OCH_3_ in ABC K+ and ABC S+ at a loading of 0.1% wt/vol. In contrast, DBS-SCH_3_ forms significantly softer, weaker gels at this loading in all products. This would suggest that the most hydrophobic gelator (DBS-SCH_3_) is somewhat less able to form a stiff, self-assembled network in these anti-icing fluids.

We then carried out temperature-dependent rheology, cooling the samples from ca. 80 °C to −5 °C on the rheometer plate and then reheating. This technique allows visualization of the onset of gel behavior on cooling as G* (complex modulus) rapidly increases, and the reconversion of gel to sol on heating as G* decreases again. It also provides insight into the hysteresis between heating and cooling. We define the temperature of gel formation *T*_f_ as the temperature at which G* reaches its maximum value, and the temperature of gel disassembly *T*_d_ as the temperature at which G* reaches its minimum value again (Table 2). This rheological method highlights subtle differences in the thermal performance of these materials that were not evident from the simple tube inversion experiments studying the visual breakdown of the bulk gel that were described above.

**Table 2:** Temperatures for Gel Formation on Cooling (*T*_f_/°C) and Gel Breakdown on Heating (*T*_d_/°C) as Determined via Variable Temperature Rheology for DBS, DBS-OCH_3_, and DBS-SCH_3_ (0.1% wt/vol), with a Strain of 0.1% and Frequency of 1 Hz

		Product		
LMWG		ABC 3	ABC K+	ABC S+
DBS	*T*_f_/°C	13.5	28.9	31.5
	*T*_d_/°C	49.1	53.8	57.9
DBS-OCH_3_	*T*_f_/°C	17.1	29.1	34.7
	*T*_d_/°C	55.2	56.6	66.1
DBS-SCH_3_	*T*_f_/°C	19.8	32.9	39.1
	*T*_d_/°C	57.9	66.1	82.1

For all LMWGs, the gels formed in ABC 3 are the least thermally stable and those in ABC S+ are the most thermally stable, with gels in ABC K+ being intermediate (Table 2, Figure 3B). This is aligned with the rheological studies described above (Table 1, Figure 3A), which indicated that the softest gels were formed in ABC 3 and the stiffest gels in ABC S+. However, in a comparison of LMWGs, DBS formed the least thermally stable gels in each case, and DBS-SCH_3_ formed the most thermally stable gels, with DBS-OCH_3_ being intermediate between the two (Table 2, Figure 3B). This is inverse to the order of stiffness observed for this family of gels, which might suggest that the slightly less ordered softer LMWG networks are thermodynamically easier to assemble. In terms of the desired application, the thermal studies demonstrated that these materials had appropriate properties for application to aircraft and were all capable of assembling into gels at temperatures >0 °C.

We then studied the kinetics of the gel assembly. Samples of LMWGs in anti-icing products were placed in an oil bath just above the *T*_gel_ value. Each sample was then applied to the rheometer plate at 20 °C to trigger the gel assembly. The time the complex modulus (G*) takes to reach a plateau was recorded as the time required for gel formation (Table 3, Figure 3C). It is evident that each gelator follows the same trend, with the time required to form a gel being ABC 3 > ABC K+ > ABC S+. Therefore, gels form most slowly in ABC 3, and the fastest in ABC S+. Perhaps surprisingly, DBS-SCH_3_ formed gels exceptionally quickly in ABC K+ or ABC S+, with the maximum G* value being reached in under 10 s.

**Table 3:** Time Required (seconds, (minutes)) for Gel Formation by DBS, DBS-OCH_3_, and DBS-SCH_3_ (0.1% wt/vol) in the Different Anti-icing Products on a Rheometer Plate Set at 20 °C (Strain 0.1%, Frequency 1 Hz)

	Time of Formation/seconds (minutes)		
	ABC 3	ABC K+	ABC S+
DBS	1210 (20.2)	810 (13.5)	640 (10.7)
DBS-OCH_3_	2675 (44.6)	1185 (19.8)	1035 (17.3)
DBS-SCH_3_	415 (6.9)	<10	<10

The rapid assembly may suggest that gel formation by DBS-SCH_3_ is kinetically controlled—indeed, in our previous work we found different assembled morphologies for DBS-SCH_3_ depending on whether kinetic or thermodynamic control was applied, leading to thin nanofibers and larger crystalline tapes, respectively. The nanoscale morphologies observed here for DBS-SCH_3_ are similar to those obtained under kinetic control. We reason that considering the fast-cooling conditions when sprayed onto a cold aircraft wing, the kinetically controlled assembly of DBS-SCH_3_ is the most relevant to the actual application.

Aging of the gels over longer periods of time was not investigated here. It is known in some cases that gels can evolve over time as samples are aged.^53^ We cannot rule such processes out but neither do we see any obvious visual evidence of gel instability. However, such studies would be important if an industrial product were ultimately brought to market to understand factors such as shelf life.

In general terms, the more effective gels formed in ABC S+ assemble more quickly than the less effective gels formed in ABC 3. Similarly, the gels based on DBS that formed stiffer networks assembled faster than those based on DBS-OCH_3_. The kinetics of gel formation are therefore broadly aligned with the rheological performance of the assembled gel network. However, gels based on DBS-SCH_3_ were surprisingly quick to assemble given their lower thermodynamic stability—this may reflect the fact that gels based on DBS-SCH_3_ are much softer, and presumably less well organized, than those based on the other LMWGs, and as such, the energy barrier to gel assembly may be significantly less.

Interestingly, the kinetics of gel formation of DBS and DBS-OCH_3_ are slower than those previously reported in a Type 1 deicing fluid with 50% MPG, where gels were typically formed within 5–8 min. In these Type II/IV fluids, gel formation instead typically takes 10–20 min (and in one case >40 min). However, it should be noted that gels formed much more quickly in a thin film on the cold stage in the WSET testing below, so this slower assembly of bulk gels is not problematic in terms of the desired application. Clearly, the presence of the polymeric additive in the Type II and IV products investigated here kinetically inhibits gel network assembly. We suggest that possible interactions between the polymer and the self-assembling gel network slow LMWG assembly. In contrast, DBS-SCH_3_ assembles exceptionally quickly in these anti-icing fluids, but the gels it forms are not particularly stiff or stable. We suggest that the enhanced hydrophobic nature of DBS-SCH_3_ leads to interactions with the polymer additives in the anti-icing formulations and that this may modify the assembly mode (see below for TEM evidence).

It is evident that in terms of overall gel assembly ABC S+ > ABC K+ > ABC 3 (Figure 3). As such, gel formation is most effective in the presence of the highest-performance anti-icing polymers, suggesting that these polymers are less disruptive to LMWG assembly. There is relatively poor gel assembly in ABC 3, which would suggest that the polymer additive in ABC 3 may have a significant impact on gel formation. Proprietary knowledge about the structure of ABC 3 leads us to suggest that DBS-based LMWGs interact noncovalently with parts of the polymer, disrupting ordered assembly. As such, this is an example in which polymer—LMWG interactions mediate the ability of the LMWG to form a self-supporting hybrid gel.

Importantly, it should be noted that in comparison to the performance of the LMWGs in base solvent described in our previous work,^40^ the LMWGs are better able to assemble in all systems that include polymer additives, indicating that even with interactive polymers, the polymer network nonetheless improves the overall ability of the system to form a sample-spanning gel. We ascribe this to the presence of the preformed polymer network acting as a scaffold, meaning the self-assembling LMWG does not have to span such large spaces to establish its own network.

**Figure 4:**
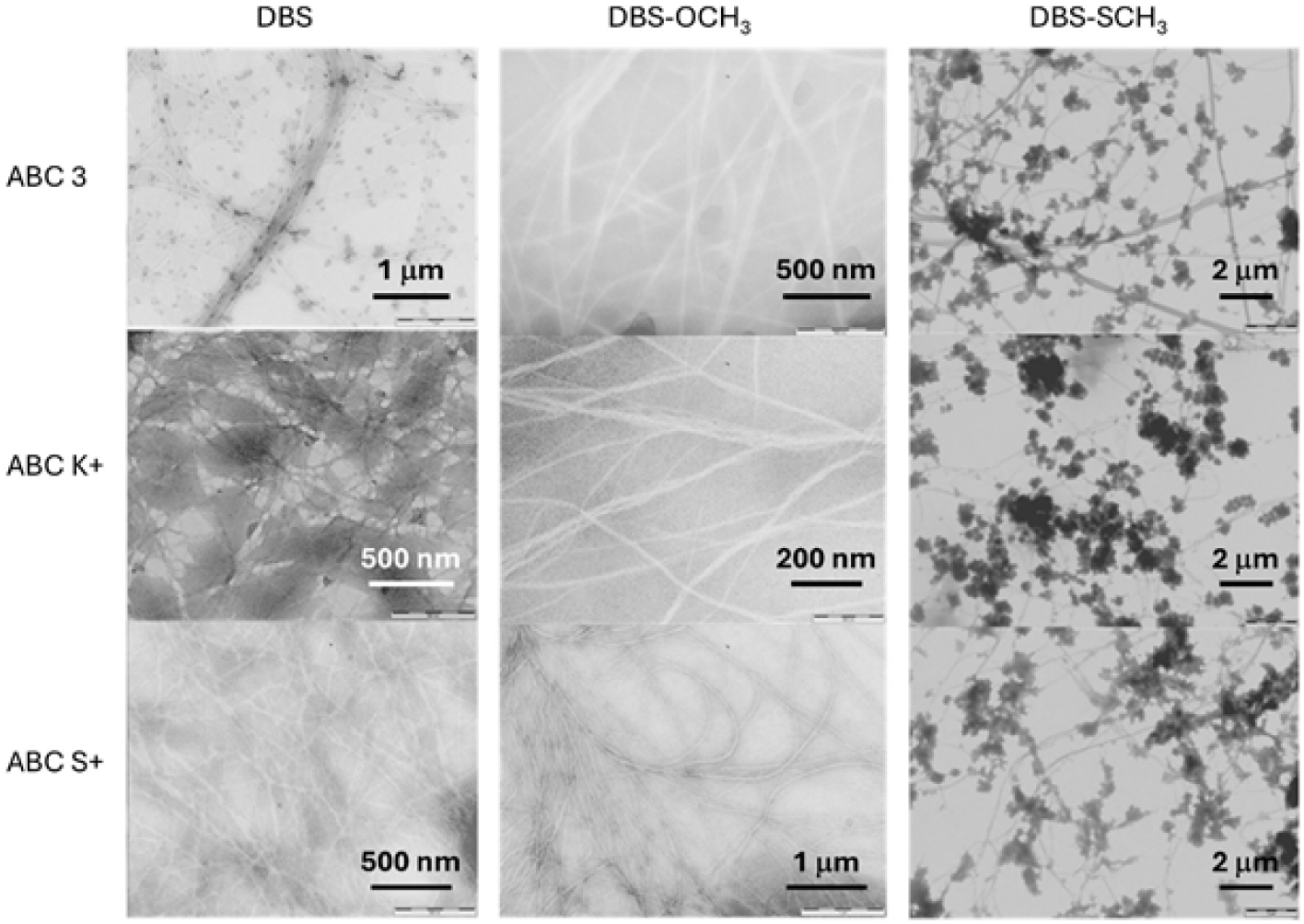
TEM images of gels formed in anti-icing fluids by different LMWG. All images show the nanofibrillar assemblies associated with the LMWG. In the case of DBS-SCH_3_, aggregates attributed to the polymeric component of the anti-icing fluid are clearly visualized attached to the self-assembled LMWG nanofibers.

### Electron Microscopy Imaging

We used scanning electron microscopy (SEM) and transmission electron microscopy (TEM) to further characterize these multicomponent materials. Initially, we studied the unmodified anti-icing fluids. Samples were prepared by adding one drop of the product onto an aluminum SEM stub or copper-backed TEM grid. This was smeared over the surface to create a thin film and dried in a desiccator. SEM and TEM were unable to visualize any nanostructures (Figure S7). We imaged the polymer dispersions added in the formulation of each anti-icing product to try and understand polymer morphology. The polymer dispersions dried very fast (<30 min), forming a film that became brittle, flaking off in areas. The polymers were very unstable under the microscope beam, but SEM indicated that the polymers form micellar structures which aggregate further (Figure S6), although it must be noted that this may be an artifact associated with the relatively high concentration of this system and the way in which it was dried. Nonetheless, it provides a useful benchmark for a comparison of samples containing the LMWGs. Although the polymers are different in each case, there are no obvious nanostructural differences. Using TEM, the samples were once again unstable under the microscope beam and difficult to image. However, it was clear that no fibers are formed, but rather small spherulitic black (negative) regions of high density were observed (Figure S8). In summary, the polymer dispersions formed nanospherical objects with no fibrillar objects being visualised.

Samples of each of the LMWGs in the three different anti-icing products were then imaged by SEM/TEM. The SEM images of samples formed in the presence of the LMWGs remained quite heavily solvated, and it was not possible to discern clear network assembly using this technique. We attempted to produce better samples using freeze-drying, but this caused the MPG to bubble and boil rather than dry smoothly, preventing us from obtaining good samples.

We therefore turned to TEM imaging of the anti-icing products in the presence of LMWGs (Figure 4). For DBS in ABC 3 we clearly visualized a fibrillar network (diameter 5–10 nm) alongside what appeared as globular structures (diameter 500 nm–3 μm). These morphologies coexist throughout the sample, with the globular structures appearing dispersed through the solvent pores and at points attached to the length of the fibers. When imaging DBS in ABC K+, we once again observed nanofibers (5–10 nm) and some higher-density darker regions, although it was less clear whether these were interconnected. Similar observations were made for gels formed in ABC S+. It should be noted that these samples were challenging to image and very unstable under the microscope beam. Nonetheless, it is evident that DBS forms the nanofibers characteristic of its gel assembly in each of the anti-icing fluids, and there is some evidence of a self-sorted hybrid network being formed alongside the polymer component, which has its own mode of assembly. DBS-OCH_3_ also clearly indicated the formation of a self-assembled nanofibrillar network within these anti-icing products (diameter 5–10 nm), although it was more challenging to image the polymer component in these hybrids.

In contrast, and in line with its different rheological performance, DBS-SCH_3_ gave very different TEM images. Once again, nanofibers were clearly observed (diameter 5–10 nm). However, on this occasion, in each product, we visualized circular globular-type structures (diameter 200 nm–1 μm) appended onto these fibers, often clumping together to create larger structures. Such objects were not observed when DBS-SCH_3_ was imaged in MPG:H_2_O in the absence of polymeric additives.^40^ Indeed, these objects are similar to the imaging of the anti-icing polymer dispersions, and we therefore assign them to the polymers within the product. In this case, there are clearly interactions between the self-assembled DBS-SCH_3_ nanofibers and the anti-icing polymer additives. We suggest that these direct interactions are responsible for the very different performance of DBS-SCH_3_ in the anti-icing fluids when compared to DBS or DBS-OCH_3_—as described above, DBS-SCH_3_ forms significantly softer, less thermodynamically stable, but kinetically fast gels. Given that DBS-SCH_3_ is the most hydrophobic of the three LMWGs tested, we propose that LMWG–polymer interactions mediated by the hydrophobic effect are responsible for these network-level interactions that destabilize the overall gel. We reason these interactions enable more rapid gel assembly (Table 3) by nucleating interactions between forming gel fibers. These interactions also make the gel more thermally stable (Table 2). However, the LMWG is then less able to organize itself to optimize the formation of a stiff self-assembled network; hence, the network remains much softer (Table 1).

### NMR Studies

To further probe the gels formed within these anti-icing fluids, we applied NMR spectroscopy. ^1^H NMR is a useful technique for quantifying mobile components within a gel, as species in the “liquid-like” phase, and hence mobile on the molecular scale, will be detected in the experiment, whereas those in the self-assembled “solid-like” network will not be observed.^54^ The use of a mobile internal standard enables quantification of the mobile components. Gels were formed in an NMR tube by cooling a hot solution of anti-icing fluid with LMWG in the presence of either acetone or DMSO (10 μL) as an internal standard. In all cases, the only peaks observed were for solvent(s) and the internal standard, with no resonances for LMWG or the polymer additive. We therefore simply conclude that, as expected from the studies above, both LMWG and the polymer component assemble into “solid-like” networks within the gel that are immobile on the NMR timescale.

### Scaling Up Gel Formation

We then scaled up the formation of these gels to a volume of 200 mL (Figure S3). Given that all the LMWGs assembled into gels at a loading of 0.1% wt/vol, we focused on this as our starting point. We also tested performance at lower loadings of 0.05% and 0.025% wt/vol. We selected these lower loadings to minimize cost but also to try and identify a sample with similar behavior to the original anti-icing product at room temperature, i.e., a viscous solution or partial gel.

On scaling up, samples required heating for longer periods (overnight) to produce transparent, homogeneous solutions. Samples were then left to fully cool under ambient conditions. We did not monitor the kinetics of gel assembly as these were somewhat variable from sample to sample, presumably dependent on differences in cooling rate and the requirement for multiple nucleation sites, variation in which will become more significant in these much larger volume samples. Furthermore, vial inversion would disrupt these gels and hence change the apparent kinetics.

DBS formed sample-spanning gels at 0.1% wt/vol, partial gels at 0.05% wt/vol, and viscous solutions at 0.025% wt/vol. In scale-up, DBS-OCH_3_ only formed partial gels at 0.1% wt/vol (unlike at a small scale, where sample-spanning gels were formed). At lower loadings, DBS-OCH_3_ behaved similarly to DBS. The fact that it was somewhat harder to form sample-spanning gels in these bulk samples is not surprising, as when testing gels by inversion, the forces exerted at the interface of a large-scale sample are significantly greater than those exerted on a small-volume sample in a vial. As such, bulk gels are less likely to form stable sample-spanning systems.

DBS-SCH_3_ behaved slightly differently in the different products. Partial gels were formed at all loadings in ABC 3. However, in ABC S+ and ABC K+, sample-spanning gels could be formed at all loadings, even at 0.025% wt/vol. We suggest that the longer heating in the scaled-up samples helps solubilize this more hydrophobic LMWG, allowing it to then self-assemble more effectively.

Based on these observations, it was evident that some degree of assembly/gelation could be achieved for all samples at a loading of 0.05% wt/vol. For further testing, we therefore selected an LMWG concentration of 0.05% wt /vol. Not only is such a low loading economically viable (it is equivalent to just 0.5 g/L), it also provided us with easily handled and applied samples. We also tested loadings of 0.025% wt/vol to determine whether the LMWG could still impact industrially relevant performance at such a low loading (0.25 g/L). It is worth noting that in this study, the LMWGs are being used at much lower loadings than in our previous work, where we modified the deicing fluid DF+ to convert it into an anti-icing product.^40^

### Water Spray Endurance Test (WSET)

We performed the industrially important water spray endurance test (WSET).^50,51^ This laboratory-based test records the “holdover performance” of fluids—i.e., the time a surface can be protected from ice buildup. The test is performed in a temperature-controlled chamber (air temperature: −5 °C). An aluminum frosticator plate that represents the leading edge on an aircraft wing is tilted at an angle of 10°. This plate has six test panels, four of which are used for sample loading, while the outer two are used as controls. A motor travels over the frosticator, spraying a fine mist of water, which on freezing produces 5 ± 0.2 gdm^–2^h of ice (the “catch”), replicating typical freezing conditions. Samples are poured along the top of the panel, fully covering the leading edge—ice formation that initiates at the top of the plate and progressively moves down. Once the first shard of ice is 2.5 cm from the top of the plate, the time is recorded, and the “endurance time” relative to the catch is hence calculated. For a fluid to be classified as an anti-icing fluid, the SAE ASTM1428 standard states that it must be able to meet specific minimum time limits (Type II, 30 min, Type III, 20 min, Type IV, 80 min, Table S2).^50^ Anti-icing fluids are commonly used undiluted, which we describe here as “100%” (although it has a 50:50 MPG:H_2_O solvent composition). However, they can also be diluted with water to yield either 75:25 product:water or 50:50 product:water dilutions. Regulations state that when diluted at 75%, Type II and IV anti-icing fluids must provide minimum endurance times of 20 min, and at 50% dilution, the endurance times must exceed 5 min (Table S2). Diluted samples provide shorter holdover due to the lower content of MPG— indeed “50%” dilutions of these products contain only 25% MPG.

Scaled-up gel samples were used for the WSET studies. In each case, a 100 mL sample of the undiluted anti-icing products, loaded with 0.05% wt/vol or 0.025% wt/vol LMWG, was used without any further preparation. The remaining 100 mL of each sample was diluted with hard water to create a 50% dilution of the product. Control samples of standard anti-icing fluid (and diluted standard anti-icing fluid) were run alongside each sample. The data from the WSET studies are presented in Table 4 and Figure 5. All of the undiluted anti-icing products meet the specified requirements, with ABC 3 < ABC K+ < ABC S+ in terms of holdover protection, as would be expected. This is why ABC 3 and ABC K+ are classified as Type II products (required minimum endurance time: 30 min), while ABC S+ is a Type IV product (required minimum holdover time: 80 min).

**Table 4:** Water Spray Endurance Test (WSET) Results for DBS, DBS-OCH_3_, and DBS-SCH_3_ in Neat Anti-icing Fluids ABC 3, ABC K+, and ABC S+

	Loading (% wt/vol)	ABC 3	ABC K+	ABC S+
No LMWG	N/A	33.4 ± 1.3	68.2 ± 2.6	90.7 ± 3.5
DBS	0.05%	53.0 ± 2.0	113.7 ± 4.4	97.2 ± 3.7
	0.025%	37.8 ± 1.5	116.6 ± 4.5	118.1 ± 4.5
DBS-OCH_3_	0.05%	38.4 ± 1.4	84.6 ± 3.3	92.4 ± 3.6
	0.025%	36.2 ± 1.4	87.1 ± 3.4	108.6 ± 4.2
DBS-SCH_3_	0.05%	50.7 ± 1.3	104.8 ± 4.0	102.7 ± 4.0
	0.025%	46.2 ± 1.8	106.1 ± 4.1	121.6 ± 4.7

**Figure 5:**
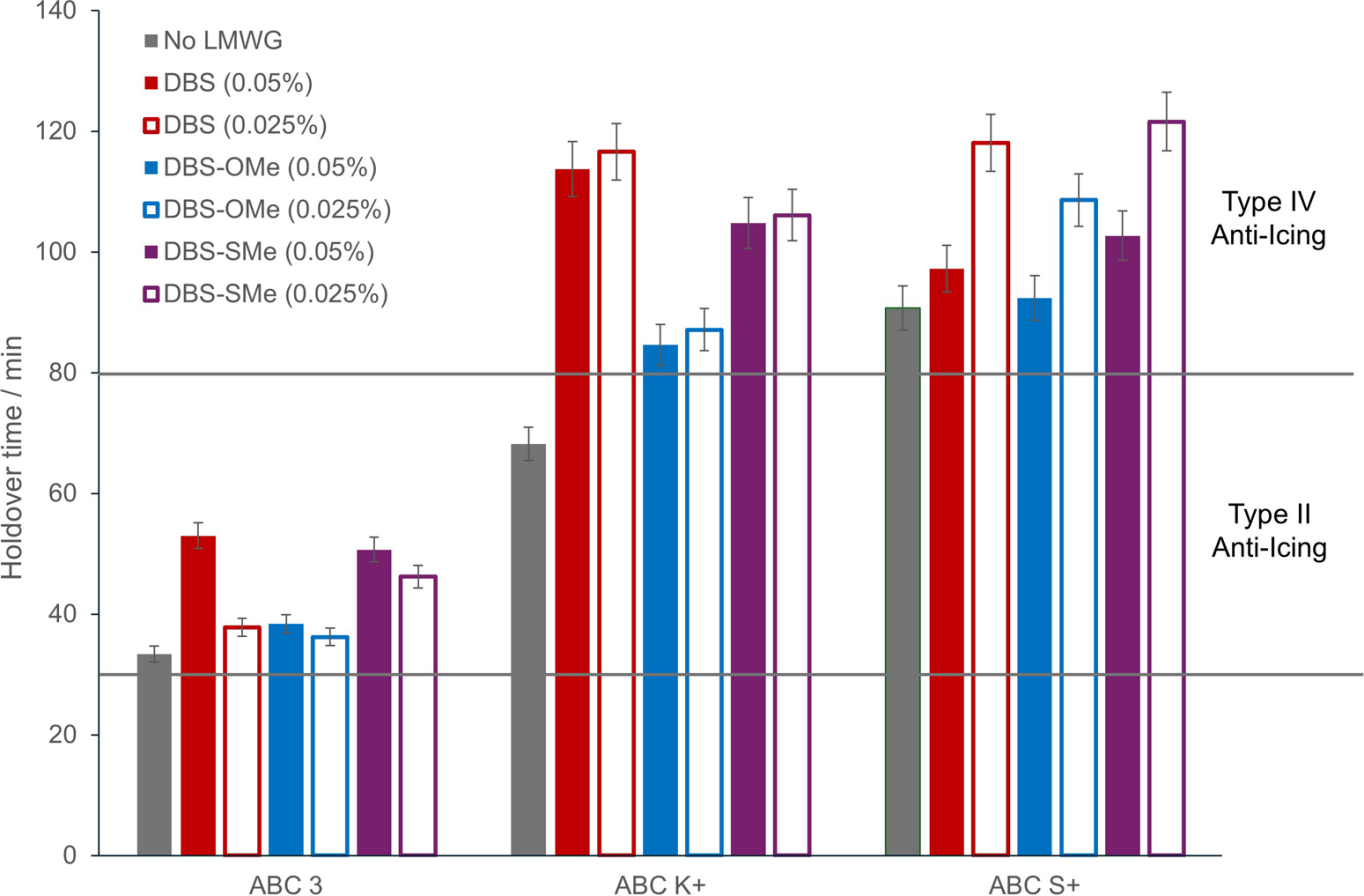
Summary of the performance of different LMWGs in anti-icing fluids using the WSET method to determine the endurance time. All gelators improve the performance of all anti-icing fluids—this is most significant for DBS and DBS-SCH_3_. At very low loadings of just 0.025% wt/vol (0.25 g/L), all three LMWGs convert ABC K+ from a Type II anti-icing fluid to a Type IV anti-icing fluid.

Pleasingly, when we add DBS into each of the anti-icing products, we increase protection, even though it is only being added at very low loadings. Indeed, adding just 0.05% wt/vol of DBS increases endurance time by 19 min in ABC 3, 45 min in ABC K+, and 7 min in ABC S+. On reducing the concentration of DBS to 0.025% wt/vol, we still increase the endurance time by 4 min in ABC 3, 48 min in ABC K+, and 28 min in ABC S+. Adding DBS to an anti-icing product clearly allows for the creation of a hybrid gel network, which improves the endurance time of each of the products. The fact that DBS has the least impact in ABC 3 is consistent with the observations above that the least effective gels are formed in this anti-icing fluid. As discussed above, this polymer is most likely to disrupt the self-assembly of the LMWG as a result of LMWG–polymer interactions. The biggest enhancements in performance are seen for ABC K+, which, in the presence of even very small amounts of DBS, is converted from a Type II product to a high-performance Type IV product. In ABC S+, the enhancement in performance provided by DBS is more significant at lower concentration, which may suggest that for this anti-icing fluid, which has an effective and well-established polymer network, too much LMWG is less desirable. Overall, given the enhancement in performance, particularly of ABC K+, combined with the low cost of DBS, and the very small amounts of LMWG being used, this is a potentially easy way of improving the performance of this class of anti-icing fluid by making a relatively small change to the overall formulation.

When using DBS-OCH_3_ as the LMWG, trends similar to those for DBS were observed. There were relatively small increases in endurance time for ABC 3, but more significant increases for ABC K+ (15–20 min) converting it into a Type IV fluid. For high-performance ABC S+, the improvement in endurance time (18 min) is once again greater at lower concentrations. In general, however, DBS-OCH_3_ is less effective than DBS in improving endurance time, which may reflect the fact that it is a slightly more hydrophilic LMWG, and possibly less able to inhibit ice formation once assembled into its sample-spanning network.

For DBS-SCH_3_, very significant improvements in the endurance time were observed. This was perhaps surprising given that this LMWG was less effective at forming gels at 0.1% wt/vol in these anti-icing fluids, and generally formed softer, less stiff gel networks. However, this indicates that protection from ice formation does not necessarily correlate with gel-forming ability. Indeed, these good results may reflect the greater hydrophobicity of DBS-SCH_3_ and the fact that this LMWG was able to form gels with lower minimum gelation concentrations (MGCs) than the others. Specifically, at 0.05% wt/vol, DBS-SCH_3_ improved the endurance time of ABC 3 by 17 min, ABC K+ by 36 min, and ABC S+ by 12 min. At 0.025% wt/vol, the endurance time of ABC 3 increased by 13 min, ABC K+ by 38 min, and ABC S+ by 31 min. Once again, the greatest improvement in endurance time was observed for ABC K+, which is converted from a Type II fluid to a high-performance Type IV product.

We suggest that the fact that all LMWGs offer the most improvement to ABC K+ reflects the balanced structural properties of this mid-performance polymer additive, which allows the LMWG both to effectively assemble in its presence while also working alongside it to inhibit the formation of ice in the optimum way. Remarkably, even at very low loadings of 0.025% wt/vol, which corresponds to just 0.25 g/L, all the LMWGs convert ABC K+ from a Type II product into a Type IV product in terms of holdover performance (Figure 5).

We then tested the three LMWGs in the diluted anti-icing fluids (Table 5). As control experiments in the absence of LMWGs, all of the diluted fluids exceeded the minimum endurance time required for a 50% diluted anti-icing product (5 min). Incorporating DBS (0.05% wt/vol) into the formulation had very little effect on the performance of ABC 3; however, the endurance time of ABC K+ was increased by 3 min and that of ABC S+ by 5 min. DBS also improved the performance of ABC K+ and ABC S+ at a loading of 0.025% wt/vol, although to lesser extents (ca. 1 and 2 min respectively). Similar trends were observed for DBS-OCH_3_ in 50% dilutions, with ABC 3 showing no improvement in endurance time, while DBS K+ was increased by 3 min and ABC S+ by ca. 2 min (0.05% wt/vol DBS-OCH_3_). Of all the LMWGs, DBS-SCH_3_ showed the most significant impact on holdover time in these diluted fluids. At a loading of 0.05% wt/vol it increased the endurance time of ABC 3 by 2 min, ABC K+ by 4 min, and ABC S+ by 5 min. At lower loading, the increases in holdover were less, except for ABC S+, which improved its endurance time by ca. 10 min. This good performance of DBS-SCH_3_ in the diluted anti-icing products mirrors its performance in the neat fluids and likely reflects its more effective hydrophobically driven assembly at low loadings, enabling it to better form a fibrillar self-assembled network and hence resist ice crystallization. In summary, however, all of the LMWGs improve the performance of the diluted anti-icing products even further above the required threshold and can therefore be considered acceptable.

**Table 5:** Water Spray Endurance Test (WSET) Results for DBS, DBS-OCH_3_, and DBS-SCH_3_ in “50%” Diluted Anti-icing Fluids ABC 3, ABC K+, and ABC S+

	Loading (% wt/vol)	ABC 3	ABC K+	ABC S+
No LMWG	N/A	6.2 ± 0.3	5.4 ± 0.2	9.6 ± 0.4
DBS	0.05%	6.7 ± 0.3	8.5 ± 0.3	14.9 ± 0.6
	0.025%	6.9 ± 0.3	6.7 ± 0.3	11.2 ± 0.4
DBS-OCH_3_	0.05%	6.9 ± 0.3	8.3 ± 0.3	11.1 ± 0.4
	0.025%	6.7 ± 0.3	6.2 ± 0.2	11.2 ± 0.4
DBS-SCH_3_	0.05%	8.1 ± 0.3	9.0 ± 0.4	14.6 ± 0.6
	0.025%	7.8 ± 0.3	6.9 ± 0.3	19.2 ± 0.7

### Aerodynamic Testing

Anti-icing products should meet an aerodynamic test to determine the ability of the fluid to flow off the aircraft during acceleration and takeoff. We thus applied rheology to test the breakdown of these gels under shear. This study was based on the AS9000 standard,^55^ stating that anti-icing fluids should be broken down by >74% of their original viscosity. A sample was applied to the rheometer plate as a hot solution, with a gel being formed *in situ*. This was tested using a strain in the LVR to ensure no damage and gel stiffness (*G*′) was determined. Increased strain was applied (10%, 50%, or 100%), with 100% strain being most similar to that experienced during aircraft takeoff to measure the breakdown of the gel into a sol. Finally, the strain was lowered again to the original value to monitor the healing of the gel. This experiment does not completely replicate the aerodynamic acceptability test but provides a useful benchmark. In particular, this approach lacks the use of fast airflow, which can remove the fluid from the surface after gel breakdown. This rheology is being performed at lower LMWG loadings than the rheology described earlier in the paper, so *G*′ values cannot be directly compared.

When using ABC 3 as the anti-icing fluid, all the samples form relatively soft networks (as described above). These are all broken down by the application of strain, with G′ values decreasing by ca. 60–70% of their original values. In the conditions of the rheology experiment, without any airflow, these gels reassemble on removal of strain, recovering 70–80% of their original *G*′ value (Figure S6A, Table S3), with the gels being formed not aging further over time. Overall, the aerodynamic testing suggests that these gels may be appropriate for use on aircraft, although their performance is borderline. Furthermore, given that the improvement in holdover times of ABC 3 induced by the LMWGs was not very significant, this is not the most promising system for further development.

For ABC K+, the gels formed using 0.05% wt/vol LMWG are somewhat stiffer than those in ABC 3 (as described above). These gels were more effectively broken down on the application of shear, with 75–80% of the original gel network stiffness being lost upon the application of high strain (Figure S6B, Table S4). On removal of strain, the hybrid gel networks regain 80–96% of their original *G*′ value. As such, the results of the aerodynamic testing are promising, and combined with the excellent WSET results in this fluid, we suggest that this system is suitable for further development.

For ABC S+, the gels formed are stiffer than those formed in either ABC 3 or ABC K+ (as described earlier), particularly for DBS and DBS-OCH_3_. Upon application of high strain, the gel networks are broken down with the *G*′ values being reduced by 80–90% (Figure 6C, Table S5). Once the strain is removed, the gel network reforms, with 90–95% of the *G*′ value being recovered for DBS and DBS-SCH_3_. However, the gel based on DBS-OCH_3_ was less able to recover (ca. 70%). It was evident that gel recovery was slower for DBS and DBS-SCH_3_ in this particular fluid, with the *G*′ rising slowly over a period of ca. 20 min, which would support the view that steric hindrance from this polymer additive somewhat limits the assembly of the LMWG in this Type IV product and introduces a degree of aging effect into the gel.

**Figure 6:**
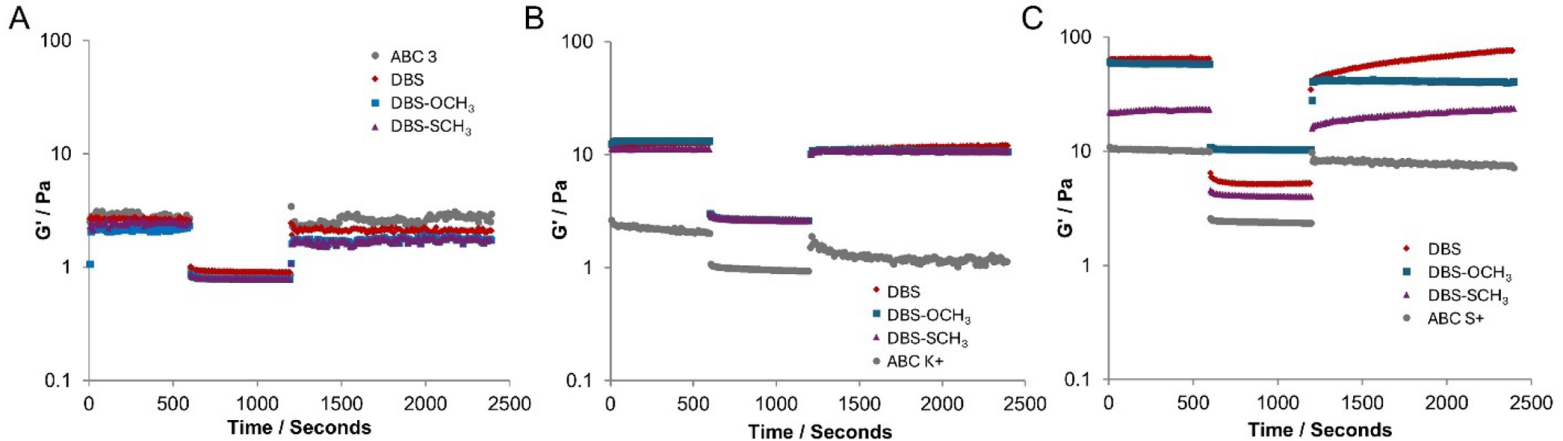
Rheology experiments on (A) ABC 3, (B) ABC K+, and (C) ABC S+, in which strain is initially within the LVR, and then increases to 10%, 50%, or 100% after 10 min. Strain is applied for 10 min, and the extent to which the gel is broken down is monitored by the decrease in *G*′. The strain is then reduced to a value within the LVR, and the ability of the gel to reform (self-heal) over time is measured. Experiments are performed in the neat anti-icing fluids (gray circles) and in the presence of LMWGs at a loading of 0.05% wt/vol: DBS (red diamonds), DBS-OCH_3_ (blue squares), DBS-SCH_3_ (purple triangles).

Overall, the aerodynamic performance of the LMWG-modified fluids was considered acceptable, particularly in ABC K+ and ABC S+ anti-icing fluids.

## Conclusions

In conclusion, we have demonstrated the ability of commercially relevant LMWGs (DBS, DBS-OCH_3_ and DBS-SCH_3_) to form gels in complex anti-icing fluids, which contain polymeric thickeners. The resulting multicomponent gels have potential use as anti-icing fluids. Gels are formed at lower concentrations than in the base solvent (MPG:H_2_O, 50:50), which suggests that the LMWG and polymeric additive cooperate in the formation of a gel network. In general, stiffer, more thermodynamically stable and more kinetically accessible gels are formed in anti-icing fluids that contain the higher-performance polymer additives (ABC S+ > ABC K+ > ABC 3). We suggest that interactions between the polymer in ABC 3 and the LMWG may compete with the assembly of these hydrophobic LMWGs, somewhat limiting their ability to form an effective sample-spanning network in this anti-icing fluid.

TEM imaging indicated the formation of a nanoscale fibrillar network for all LMWGs in these anti-icing fluids. However, in the case of the most hydrophobic gelator, DBS-SCH_3_, we could also clearly visualize globular assembled structures associated with the polymeric additive attached to these gel fibers. This correlated with the fact that of the LMWGs, DBS-SCH_3_ gave the least stiff, least thermally stable gels, suggesting that interaction between the polymeric additive and the hydrophobic LMWG disrupts the network-forming ability of DBS-SCH_3_. Scaling-up the samples indicated that in general, bulk, sample-spanning gels were somewhat more difficult to form, as might be expected given the relatively low *G*′ values of these gels and the significant forces exerted on inversion of a bulk gel sample. However, self-assembling materials were generated suitable for testing as enhanced anti-icing fluids.

Hybrid gels were tested as anti-icing agents using the WSET method, with increases in holdover times being achieved in all cases and very significant improvements in some. Most pleasingly, the performance of the undiluted Type II fluid ABC K+ could be improved to that of a high-performance Type IV fluid by the addition of very small amounts of LMWG (0.025% wt/vol). The impact of LMWGs on the WSET performance of ABC 3 was less significant, which we ascribed to the interactive nature of the polymeric additive in this fluid inhibiting such effective gel assembly. For ABC S+, the most effective additive was DBS-SCH_3_. It is interesting to reflect that this is the most hydrophobic gelator, and it is possible that the introduction of additional hydrophobicity into this product plays a key role in helping limit the buildup of ice. We also demonstrated improvements in performance in the diluted anti-icing products. Aerodynamic testing indicated that the gels in ABC K+ and ABC S+ had acceptable profiles and could be broken down by shear strain.

Remarkably, even at very low loadings of 0.025% wt/vol, which corresponds to just 0.25 g/L, all of these LMWGs convert ABC K+ from a Type II product into a high-performance Type IV anti-icing product. Given the low cost of DBS, which is a commodity chemical, it is anticipated that its use in the formulation of anti-icing fluids may be a cost-effective approach to maximizing performance.

In summary, these LMWGs have potential as formulation agents for improving the performance of complex anti-icing fluids. Given the low cost of these LMWGs, their potential to be synthesized at scale, and the ease of coformulation, we suggest that these simple self-assembling systems could see application in this type of technology. Furthermore, this approach opens the longer-term possibility of moving anti-icing technology beyond polymer-based systems and toward a more sustainable future based on reversible, self-assembling molecular materials.

## Supplementary Material



## Notes

Additional characterization data (PDF)
