# Supplementary material for: Self-Assembly of Supramolecular Gels in Complex Anti-icing Fluids to Create Multicomponent Materials with Enhanced Performance
Source: Langmuir. 2025 Dec 5;41(49):33491–503. doi: 10.1021/acs.langmuir.5c05067 (PMC12713764; doi:10.1021/acs.langmuir.5c05067)
Supplement: Supplementary file 1 [file la5c05067_si_001.pdf]

Supporting Information

Self-Assembly of Supramolecular Gels in Complex Anti-Icing Fluids to Create Multi-Component Materials with Enhanced Performance

*Nicole K. McLeod,[a,b] Lee Stokes,[b] Jerry Lewis,[b] and David K. Smith *[a]*

[a] Department of Chemistry, University of York, Heslington, York, YO10 5DD, UK.
Email: david.smith@york.ac.uk
[b] Kilfrost Ltd, Albion Works, Haltwhistle, NE49 0HJ, UK

SUPPLEMENTARY INFORMATION

S1 Experimental Methods
S2 Gel Preparation and Analysis
S3 Rheology
S4 Electron Microscopy
S5 Water Spray Endurance Test (WSET)
S6 Aerodynamic Testing Using Rheology
S7 References

S1 Experimental Methods

General Equipment. T_{gel} measurements were determined using a thermoregulated oil bath at 1°C increments. Samples for scanning electron microscopy (SEM) were prepared by spreading a small amount of gel over an aluminium stub and dried in a desiccator. They were then sputtered coated with a 4 nm layer of Au/Pd using a Polaron Agar High Resolution Sputter Coater and imaged with a JEOL JSM-7600F FEGSEM. Transmission electron microscopy (TEM) was performed on copper-backed TEM grids and left to air dry overnight and imaged using a FEI Tecnai G2 fitted with a CCD camera. Dynamic rheological measurements were performed on a Malvern Kinexus Pro+ rheometer using 20 mm parallel plate geometry and a 1 mm gap. All measurements were performed within the linear viscoelastic region (LVR) and data interpreted using rSpace for Kinexus software. The Water Spray Endurance Test (WSET) was carried out in a climatic (temperature and humidity) controlled tunnel at Kilfrost Limited. All tests were conducted under industry standard conditions at -5 °C.

Determining Minimum Gelation Concentrations (MGCs). Gels of decreasing concentrations were prepared using the standard method above. They were then inverted to identify if the gel could support

itself under gravity. The lowest concentration at which a gel was stable when inverted was taken as the MGC.

Measuring T_{gel} Values. Gel samples were used to measure the T_{gel} value. The samples were placed in a thermostatic oil bath and the temperature increased at a rate of 1.0 °C min^{-1}. As the temperature increased, the gel was removed from the oil bath at every 1 °C increase and inverted (tube inversion test). The temperature at which the gel could no longer support itself under gravity and collapses to the bottom of the vial was recorded as the T_{gel}, the temperature of the gel–sol transition. All T_{gel} values were repeated at least once and averaged.

Rheology

Preparation of Rheology Samples. For each sample a known amount of gelator was weighed out into a glass vial (10 ml) and solvent (8 ml of anti-icing product) added. The samples were sonicated for 1 hour and then heated in an oil bath to just below the boiling point of the solvent until a clear homogeneous solution was formed. The samples were left overnight to form gels at room temperature for testing the next day. As gels can be fragile if applied as a solid, the samples were applied as a solution and the gel allowed to set on the sample plate. The samples were temperature dependent, therefore each vial was placed in a water bath and heated to just above the T_{gel} value until the solution formed. Using a spoon to reduce any shear or damage to the sample, ~2 mL of the solution was placed onto the sample stage on the rheometer set to 20 °C and the geometry configured to a gap of 1 mm. The sample was trimmed to remove any excess sample and a hood placed on to the geometry to reduce evaporation before a test was started.

Amplitude Sweep. A sample was applied as above and left to equilibrate at 20 °C for 15 minutes to allow the sample to reach temperature and the gel to form. The sample was then tested across a range of increasing strain (0.001 to 100%) at a set frequency of 1 Hz and set temperature of 20 °C. This test determines the LVR (linear viscoelastic region) of each sample. A value from the LVR was then used for further tests.

Frequency Sweep. The sample was applied as above and left to equilibrate for 10 minutes on the instrument. Using a known strain value from the LVR the sample was tested across a range of frequencies (0.01 to 100 Hz) at a set temperature of 20 °C.

Variable Temperature Rheology. The samples were applied to the rheometer geometry as described above but this time to a hot sample stage set to 85 °C. The test was carried out using a known strain value within the LVR at a set frequency of 1 Hz and cooled across a temperature range (85 to -5 °C) at increments of -2 °C/min. The test was then equilibrated for 5 minutes at -5 °C before completing a heating cycle at +2 °C/min from -5 to 100 °C. Temperature ramps provide information on the behaviour of gel samples to both decreasing and increasing temperature and allow determination of $T_{sol\text{-}gel}$ (the onset of gel formation, onset-T_f was most easily determined here and $T_{gel\text{-}sol}$ (complete gel dissolution, T_d).

Time-Resolved Rheology. Each sample was applied hot to a 20 °C sample stage and the test started as quickly as possible. The test uses a known strain within the LVR at a set frequency of 1 Hz at a set temperature, 20 °C, over 1 hour to characterise how fast a gel can form under given fixed conditions.

Electron Microscopy

Scanning Electron Microscopy. Gels were formed using the standard method (see above). Once the gels had set, a small amount of gel was removed with a spatula and spread thinly onto an aluminium SEM stub. These were then placed on a polystyrene holder and placed in a desiccator to air dry for 2 days to 2 weeks depending on the solvent, to leave the xerogel. The xerogel was coated with a 4 nm layer of Au/Pd using a Polaron High Resolution Sputter coater and imaged using a JEOL 7600F FEG-SEM.

Transmission Electron Microscopy. TEM was only carried out on polymer dispersions. A small amount of polymer was placed on a copper backed TEM grid and left to air-dry overnight. The samples were then imaged using a FEI Tecnai G2 fitted with a CCD camera.

Water Spray Endurance Testing (WSET)

The water spray endurance test is a laboratory-based test developed to evaluate the endurance (holdover) performance of anti-icing fluids under freezing conditions.[50,51] This test models the length of time an aircraft has between application of an anti-icing fluid to taxiing and take-off before reapplication is required due to further ice contamination. This endurance testing was carried out within a temperature-controlled climatic chamber, and conducted at -5 °C. Within this chamber, an aluminium frosticator plate, representative of an aircraft's leading edge on a wing was setup at -5 °C with a 10° angle. The frosticator consists of 6 test panels. Four panels were used for test samples with the remaining two acting as controls, with the use of aluminium square plates which are weighed before and after the test to identify the weight of ice formed throughout the test known as the "catch". The weight of the ice ("catch") formed during the test was ~5 ± 0.2 $g/dm^2\ h^{-1}$ as detailed in AS5901.

Each sample (75 ml) was applied to the top of the aluminium test panels by pouring the fluid from left to right ensuring the top lip (leading edge) was covered. The sample flowed down the test panel through gravitational forces and wet the panel. This was repeated for every sample tested. Once all samples were applied, they were then left for 5 minutes on the frosticator plate to reach temperature. After 5 minutes, the test was started by turning on the motor above the frosticator plate which holds a spray nozzle. This nozzle sprays water at a rate of 0.5 mL/min creating a fine mist which moves forward and backward over the frosticator plate covering the samples. As soon as the spray started, the time was recorded. Gravity causes the fluid to run down the plate and coat it, however, this makes the fluid thinner at the top compared to the bottom. As the test progresses frost forms from the top edge of the panels and works its way downwards. At 25 mm from the top edge is a line indicating the end of the test zone. As soon as the first shard of ice touched this line, the time was recorded and used to calculate the endurance time for each sample. Each sample was treated in this way and when all samples were complete the test was stopped and the end time recorded. The control plates were weighed after the test and used to calculate the catch. Using the catch, end time of experiment, and the completion time for

each sample, holdover values were calculated. All WSET tests were carried out twice and the results averaged.

Anti-icing samples made with gelators (and 50% diluted samples) as described above were applied at room temperature. This is because anti-icing fluids are typically applied cold, unlike the de-icing products described in our previous research,[40] which are applied hot. Control samples of the original anti-icing products undiluted and at 50:50 dilution were run alongside the samples containing LMWG as a control. WSET was carried out for each of the different anti-icing products with each of the different LMWGs.

Aerodynamic Testing using Rheology

To determine the effects of shear on gel samples and to identify if these gels would break down and be able to be removed from an aircraft after takeoff, variable shear rheological analysis was performed. Samples were applied hot to the rheometer stage, with the stage set at 20 °C. Each sample was set to equilibrate for 15 minutes before the test was started, to allow the gel to form. This test was then set to use a strain within the LVR with a frequency of 1 Hz at 20 °C for 10 minutes. The parameters were then changed to apply a strain of 10% (outside of the LVR) for 10 minutes with the remaining parameters staying constant. Finally, the strain was returned to the initial value for 1 hour. These steps were run consecutively with no breaks in between. This characterises the ability of the gel to set up on a surface, be broken down and then finally recover ('self-heal'). To further understand the effects of increasing strain, the test was repeated twice more for each sample applying 50% or 100% strain in the second step of the experiment.

S2 Gel Preparation and Analysis

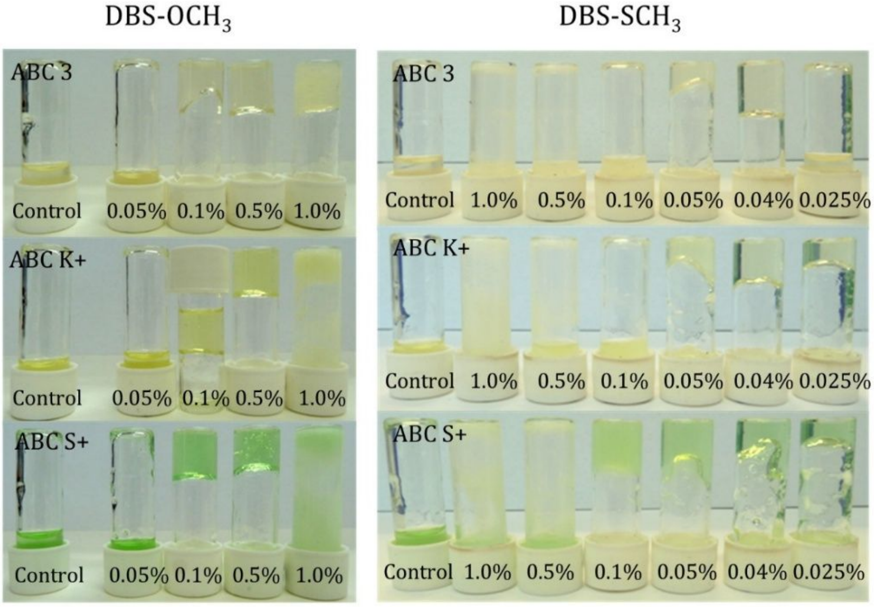

Figure S1. Photographs of gels formed by DBS-OCH_3 (left) and DBS-SCH_3 (right) in anti-icing fluids ABC 3 (top), ABC K+ (centre) and ABC S+ (bottom) at different loadings from 0.025 to 2% wt/vol.

Table S1. MGC values (% wt/vol) of the gelators in Type II and Type IV Anti-icing products.

**Gelator**	**Minimum Gelation Concentration % wt/vol**		
	**ABC 3**	**ABC K+**	**ABC S+**
**DBS**	0.06	0.06	0.05
**DBS-OCH_3**	0.06	0.06	0.06
**DBS-SCH_3**	0.04	0.04	0.04

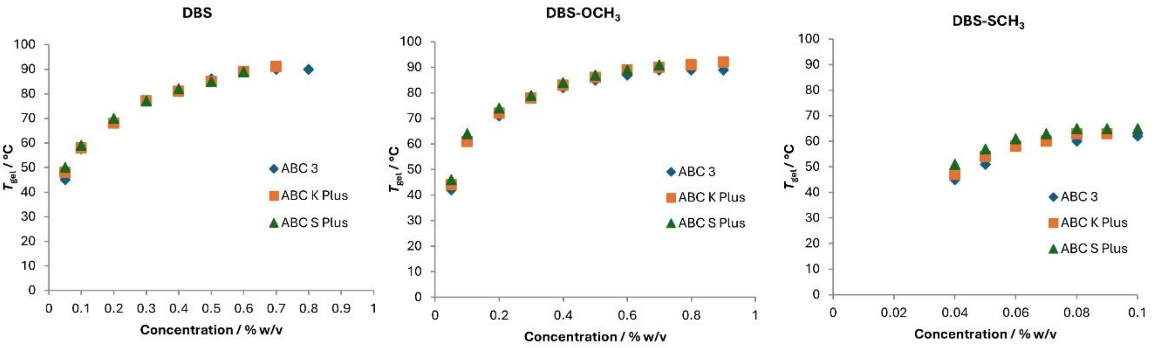

Figure S2. Concentration dependent T_{gel} values in (left) DBS, (centre) DBS-OCH_3 and (right) DBS-SCH_3) in different anti-icing fluids (blue diamonds = ABC 3, orange squares = ABC K+, green triangles = ABC S+).

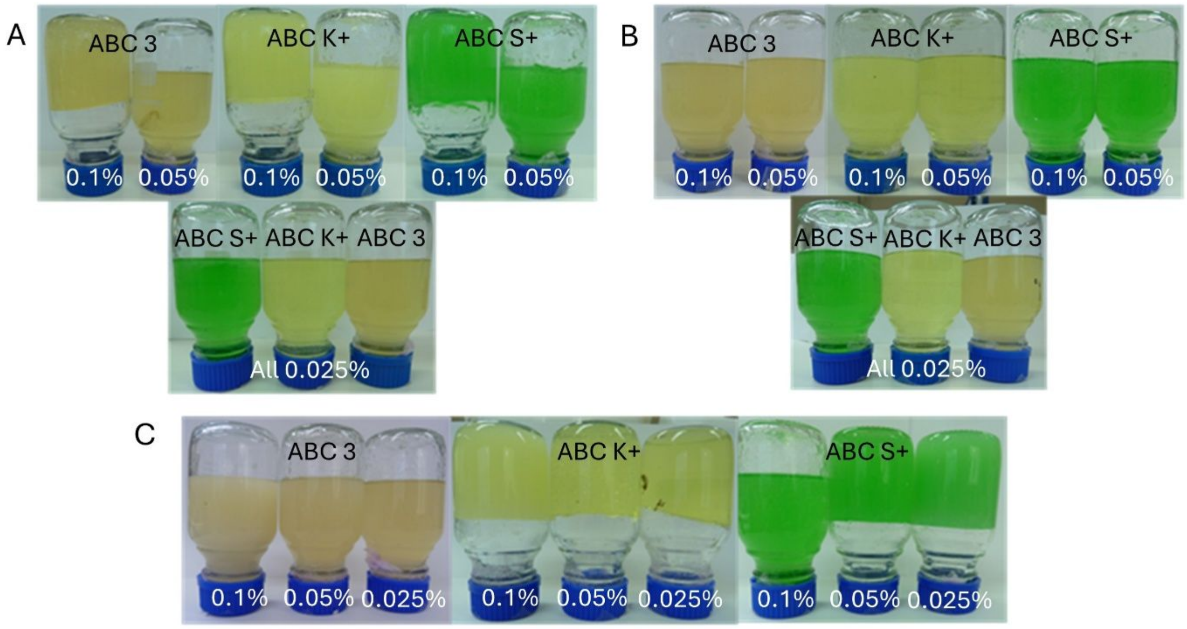

Figure S3. Photographs of bulk gels formed by (A) DBS, (B) DBS-OCH_3 and (C) DBS-SCH_3 at loadings of 0.1%, 0.05% and 0.025% in ABC 3 (straw), ABC K+ (yellow), ABC S+ (green).

S3 Rheology

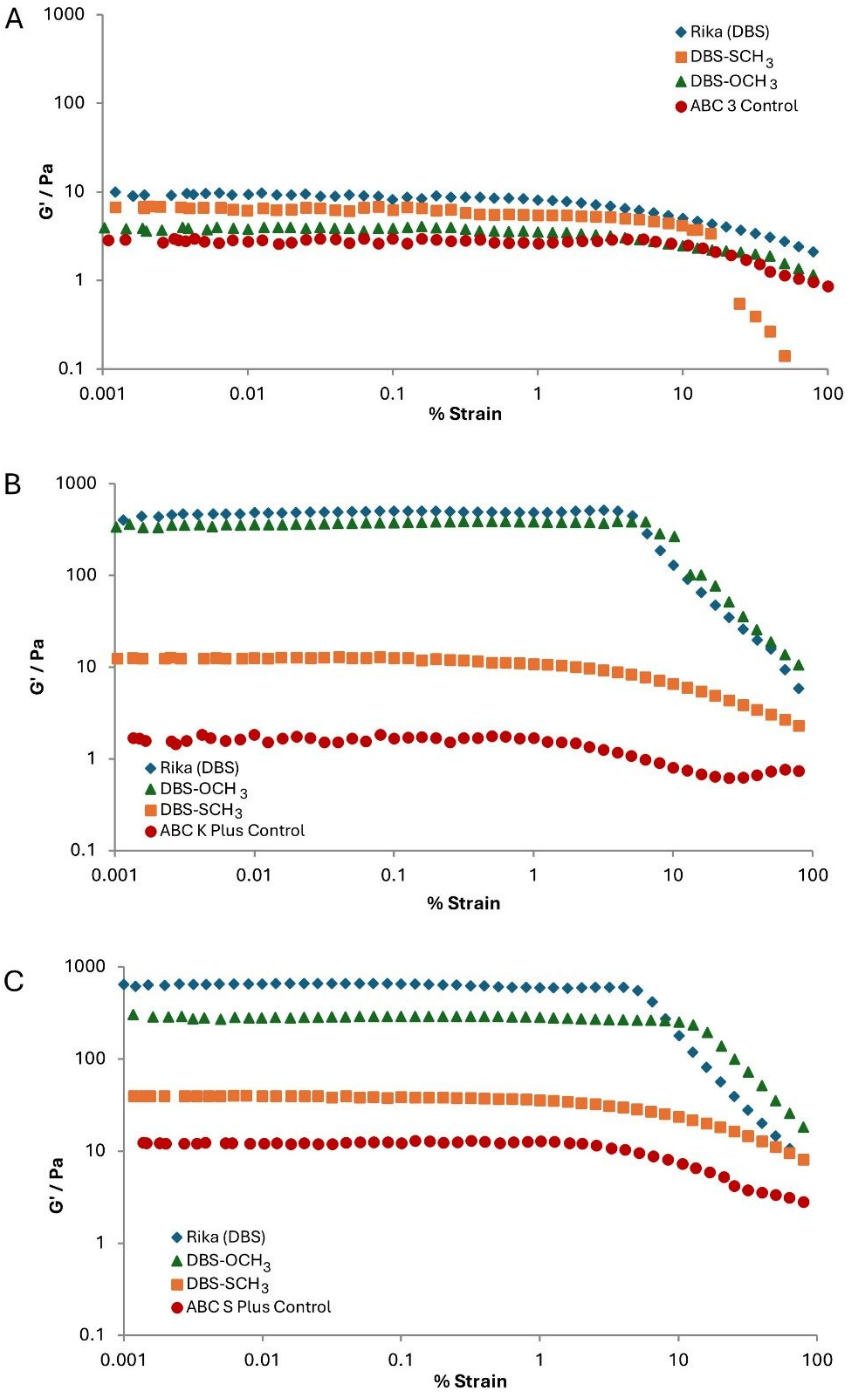

Figure S4. Amplitude sweep rheometry in (A) ABC 3, (B) ABC K+ and (C) ABC S+ for DBS (dark blue diamonds), DBS-OCH_3 (green triangles) and DBS-SCH_3 (orange squares) all at loadings of 0.1% wt/vol, with the rheometry of the individual anti-icing fluids as a control (red circles).

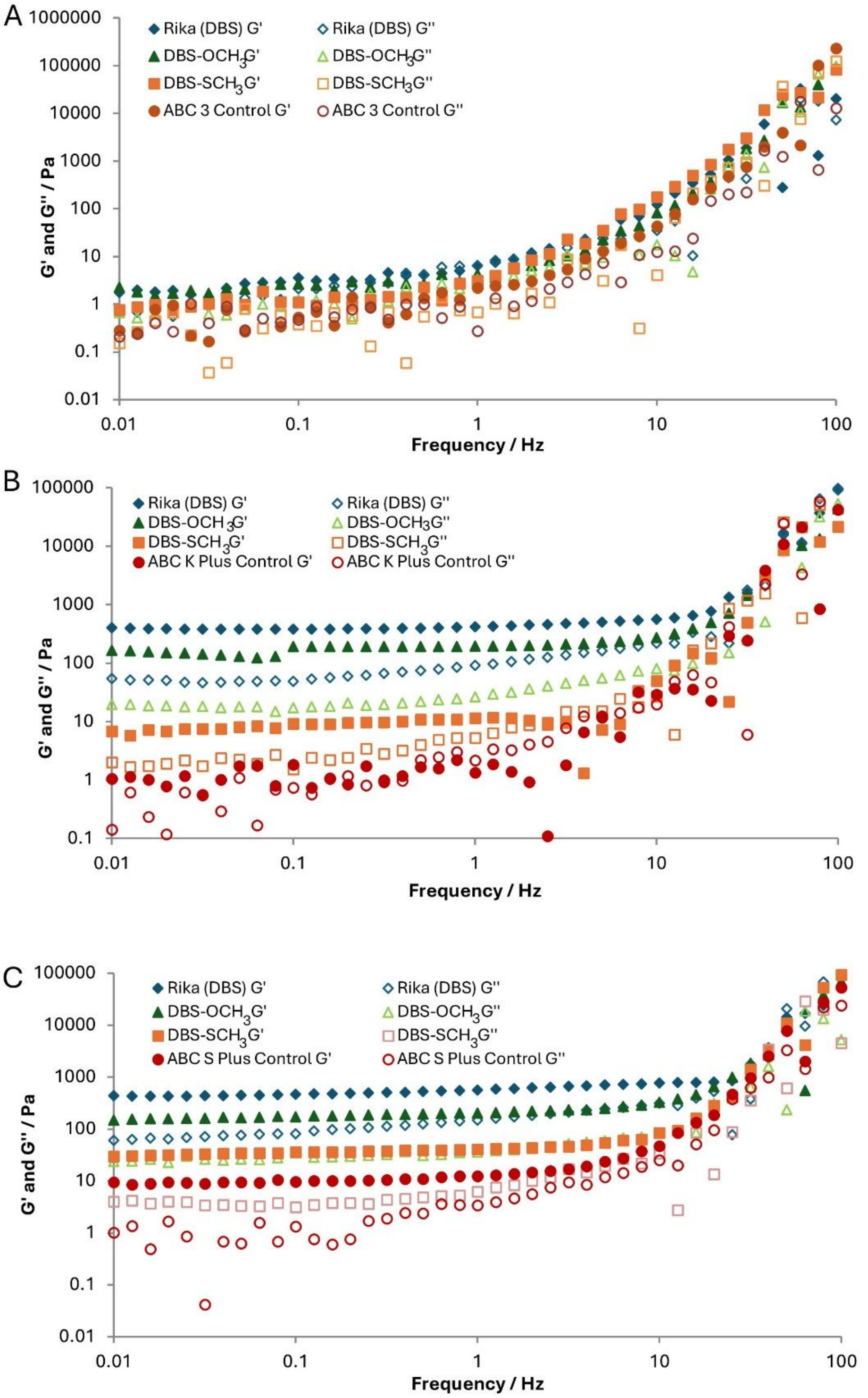

Figure S5. Frequency sweep rheometry in (A) ABC 3, (B) ABC K+ and (C) ABC S+ showing G' (closed shapes) and G" (open shapes) for DBS (dark blue diamonds), DBS-OCH_3 (green triangles) and DBS-SCH_3 (orange squares) all at loadings of 0.1% wt/vol, with the rheometry of the individual anti-icing fluids as a control (red circles).

S4 Electron Microscopy

S4.1 Scanning Electron Microscopy (SEM)

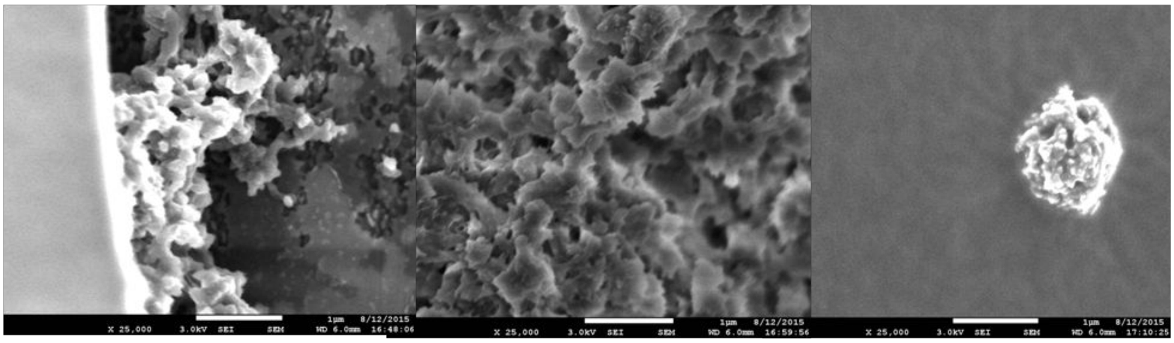

Figure S6. SEM imaging of the three different polymer dispersions found within each of the anti-icing products, ABC 3, ABC K Plus and ABC S Plus.

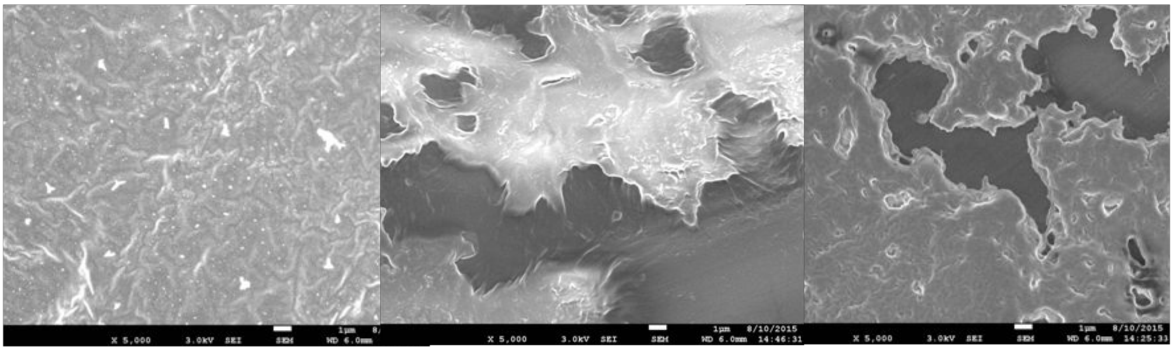

Fig. S7. SEM analysis of Rika (DBS) in each of the anti-icing products, ABC 3, ABC K Plus and ABC S Plus.

S4.2 Transmission Electron Microscopy (TEM)

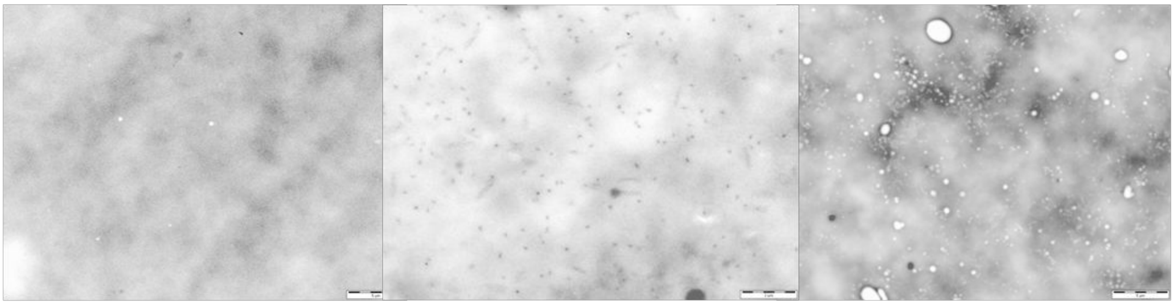

Figure S8. TEM analysis of the three different polymer dispersions used within anti-icing products ABC 3, ABC K Plus and ABC S Plus.

S5 Water Spray Endurance Test (WSET)

Table S2. Minimum WSET 'holdover' times as specified by the relevant regulator.

Dilution	Minimum WSET Holdover Time (minutes)		
	Type II	Type III	Type IV
**100%**	30	20	80
**75%**	20	Report	20
**50%**	5	Report	5

S6 Aerodynamic Testing using Rheology

Table S3. Percentage of fluid removed after high strain and the percentage recovery of each of the gel networks formed with each gelator in ABC 3.

Gelator	% Decrease in G′ after application of high strain	% Recovery of G′ relative to original value after removal of strain
DBS	66	80
DBS-OCH_3	63	80
DBS-SCH_3	68	70
ABC 3 Control	69	92

Table S4. Percentage of fluid removed after high strain and percentage recovery of each gel network for each gelator in ABC K Plus

Gelator	% Decrease in G′ after application of high strain	% Recovery of G′ relative to original value after removal of strain
DBS	80	82
DBS-OCH_3	78	93
DBS-SCH_3	76	96
ABC K+ Control	57	56

Table S5. Percentage of fluid removed after high strain and percentage recovery of each gel network formed by each gelator in ABC S Plus

Gelator	% Decrease in G′ after application of high strain	% Recovery of G′ relative to original value after removal of strain
DBS	92	96
DBS-OCH_3	83	70
DBS-SCH_3	82	91
ABC S+ Control	77	77

S7 References

1. McLeod, N. K.; Stokes, L. Lewis, J.; Smith, D. K. Supramolecular Gels with Potential Applications as Anti-Icing Agents, *Langmuir*, **2025**, *41*, 13932-13947.
